# Molecular characterization of *Vibrio cholerae* responsible for cholera epidemics in Uganda by PCR, MLVA and WGS

**DOI:** 10.1371/journal.pntd.0006492

**Published:** 2018-06-04

**Authors:** Godfrey Bwire, David A. Sack, Mathieu Almeida, Shan Li, Joseph B. Voeglein, Amanda Kay Debes, Atek Kagirita, Ambrose Wabwire Buyinza, Christopher Garimoi Orach, O. Colin Stine

**Affiliations:** 1 Ministry of Health Uganda, Department of Community Health, Kampala, Uganda; 2 Johns Hopkins Bloomberg School of Public Health, Department of International Health, DOVE Project, Baltimore, Maryland United States of America; 3 University of Maryland School of Medicine, Department of Epidemiology and Public Health, Baltimore, Maryland, United States of America; 4 Uganda National Health Laboratory Services (UNHS/CPHL), Kampala, Uganda; 5 Makerere University, Department of Geography, Kampala, Uganda; 6 Makerere University School of Public Health, College of Health Sciences, Kampala, Uganda; Institut Pasteur, FRANCE

## Abstract

**Background:**

For almost 50 years sub-Saharan Africa, including Uganda, has experienced several outbreaks due to V*ibrio cholerae*. Our aim was to determine the genetic relatedness and spread of strains responsible for cholera outbreaks in Uganda.

**Methodology/Principal findings:**

Sixty-three *V*. *cholerae* isolates collected from outbreaks in Uganda between 2014 and 2016 were tested using multiplex polymerase chain reaction (PCR), multi-locus variable number of tandem repeat analysis **(**MLVA) and whole genome sequencing (WGS). Three closely related MLVA clonal complexes (CC) were identified: CC1, 32% (20/63); CC2, 40% (25/63) and CC3, 28% (18/63). Each CC contained isolates from a different WGS clade. These clades were contained in the third wave of the 7^th^ cholera pandemic strain, two clades were contained in the transmission event (T)10 lineage and other in T13. Analysing the dates and genetic relatedness revealed that *V*. *cholerae* genetic lineages spread between districts within Uganda and across national borders.

**Conclusion:**

The *V*. *cholerae* strains showed local and regional transmission within Uganda and the East African region. To prevent, control and eliminate cholera, these countries should implement strong cross-border collaboration and regional coordination of preventive activities.

## Author summary

Cholera, an acute diarrheal disease, essentially was eliminated in the western world many decades ago, but has continued to cause many deaths in sub-Saharan Africa, South America and Asia. Cholera diagnosis in most countries in sub-Saharan Africa, including Uganda, is by stool culture, serology and biochemical methods. These testing methods are unable to establish the relatedness, virulence and spread of *Vibrio cholerae* in region. To determine the spread, relatedness and virulence of *V*. *cholerae* responsible for the various cholera outbreaks in Uganda, we used DNA-based testing methods. We tested 63 *V*. *cholerae* isolates from samples collected in Uganda from 2014–2016. Our results showed three distinct lineages of genetically related cholera-causing bacteria. These organisms showed internal spread in Uganda and cross-border spread to neighboring countries in East Africa. These findings provide a valuable baseline and help define the context for directing control measures and technologies for cholera prevention in East Africa.

## Introduction

*Vibrio cholerae* remains a major cause of morbidity and mortality globally [1]. There have been seven cholera pandemics since the disease was recognized as a global threat [2]. The English record of pandemics of cholera started in 1816, but cholera as a disease goes back centuries in Indian literature [3]. The organism responsible for cholera outbreaks, *V*. *cholerae*, was cultured over 130 years ago by Robert Koch (1884) in India [4] and its epidemiology in England was described by John Snow in 1886 [5].

Over time, considerable knowledge and skills in the management of this deadly infectious disease have accumulated leading to better prevention and control of epidemics [6–8]. Industrialized countries essentially have eliminated cholera as a public health problem through improved water and sanitation [9]. Nonetheless, this enteric bacterium continues to cause deaths and suffering in many countries [10–12]. Sub-Saharan Africa bears the highest reported cholera disease burden [13]. The ongoing outbreaks in Africa and elsewhere in the world are part of the seventh pandemic caused by the *V*. *cholerae* O1, El Tor lineage [14,15]. Genetic differences among isolates allow for a greater understanding of the transmission of the bacteria within and between geographic regions and time periods [16].

Two methods, multilocus variable-number tandem-repeat analysis (MLVA) [17,18] and whole genome sequencing (WGS) [19], provide sufficient genetic differentiation to distinguish between the isolates across different places and times. Less complex methods such as culture, biochemical and serological tests to detect, confirm and describe *V*. *cholerae* [20], do not permit accurate tracking of the spread of specific genetic lineages. Yet these are the only methods available in most African countries including Uganda [21]. The goal of this study was to analyze *V*. *cholerae* isolates responsible for cholera outbreaks that occurred between 2014–2016 in Uganda using multiplex PCR, MLVA and WGS to determine the genetic relatedness and spread of *V*. *cholerae* isolates from different outbreaks in Uganda.

## Materials and methods

### Study design

A cross-sectional study was conducted using all available viable *V*. *cholerae* isolates collected during cholera outbreaks in Uganda between 2014 and 2016 and kept frozen (-80°C) at the Central Public Health Laboratory (CPHL) in Kampala. In addition, aggregated epidemiological cholera surveillance data for the years 2014–2016 were reviewed and used to generate Epi-maps that contextualized the epidemic spread and transmission of cholera.

#### Ethical considerations

Permission to conduct the study was obtained from the Makerere University School of Public Health Institution Review Board (IRB number, 00011353). The isolates were collected through the Ministry of Health disease surveillance system and stored at the CPHL. Personal identifiers were removed by labeling the isolates using the district name and district codes.

#### Data management

Data used to create the disease distribution over the period 2014–2015 were from the Uganda Ministry of Health epidemic disease surveillance system which is part of the national health management information system (S1 Dataset). Data were analyzed to calculate percentages and proportions. Aggregated cholera cases and deaths were analyzed and used to generate maps. Shapefiles used to create the Uganda maps were obtained from the Uganda Bureau of Statistics. The maps were created using the Arc View Geographical Information System (GIS).

#### Recovery of frozen isolates

*V*. *cholerae* isolates were recovered from frozen storage. During this process safety precautions were observed as described in standard laboratory manuals for epidemic dysentery and cholera diagnosis [22,23]. The recovered isolates were packaged and shipped to Baltimore, Maryland, USA, for genetic testing.

#### PCR test

To confirm the isolates as *V*. *cholerae* and to determine their virulence by PCR tests, primers targeting *ompW (*outer membrane protein*)*, *ctxA (cholera enterotoxin sub-unit A)* and *toxR* (transcription activator controlling cholera toxin) were used. DNA was extracted and amplified using primers as described previously [24].

#### Multi-locus variable tandem repeat analysis (MVLA)

DNA was genotyped for five MLVA loci: VC0147, VC0436-7 (intergenic), VC1650, VCA0171 and VCA0283 [18]. Each of the five loci was amplified as described previously [17,18]. The presence of amplified products was confirmed by gel electrophoresis.

The fluorescently labeled amplified products were separated using a 3730xl Automatic Sequencer with the size determined from internal lane standards (LIZ600) by the GeneScan program (Applied Biosystems, Foster City, CA). The genotypes for each isolate are in supplementary table 1 (S1 Table) EBURST (www.mlst.net) was used to define the genetic relatedness between genotypes. Genotypes within a clonal complex were related by a series of single locus variants.

#### Whole genome sequencing

Three or four representative samples were selected from each of the 3 MLVA clonal complexes identified during the period 2014–2016 for testing by WGS. Libraries for Illumina sequencing were prepared from DNA fragmented with Covaris E210 (Covaris, Wolburn, MA) using the KAPA High Throughput Library Preparation Kit (Millipore-Sigma, St. Louis MO). The libraries were enriched and barcoded in ten cycles of PCR amplification with primers containing an index sequence. Subsequently, the libraries were sequenced using a 100 bp paired-end run on an Illumina HiSeq2500 (Illumina, San Diego, CA).

The quality of the 101-base paired-end reads was assured by a quality trimming procedure using Sickle (v1.33), with a minimum read length after trimming of 75nt, and a quality threshold of 20. High quality reads were assembled with “Spades” software (v.3.6.2). Annotation was performed using the RAST server [25]. The annotated sequences were submitted to Genbank Accession number PYRD00000000-PYRM00000000. The BioProjectID is PRJNA439310.

Nucleotide variation was identified and compared to *V*. *cholerae* O1 El Tor strain to identify single nucleotides variants (SNVs). Parsnp (v1.2) was used to align the variable nucleotides from the core-genome using the option ‘–c’ to constrain the use of all input genomes and generate the ‘.vcf’ variant description file and ‘.ggr’ alignment description file. The ‘.ggr’ file was loaded in Gingr (v1.2) to visualize the alignments and export the variable nucleotide alignment ‘.mfa’ file [26]. The ‘.vcf’ file was then used to remove all variable nucleotides from the ‘.mfa’ file detected near the edge of the contigs (less than 1 kb of the contigs edges) using an in-house script. Information about each genome sequence is in Supplemental Table 1 (S1 Table). No regions with an excess density of SNPs were detected.

To understand the relatedness of the Ugandan strains to those from the seventh pandemic, 41 representative African isolates with known WGS from the wave 3 transmissions T10, T11 and T12 were selected (S2 Table) and analyzed with the Ugandan sequences in FastTree2 (v2.1.9) [27] with default parameters to generate the maximum-likelihood tree. Data were displayed and visualized using Interactive Tree of Life (iTOL) [28].

## Results

A total of 63 *V*. *cholerae* isolates for the years 2014–2016 were tested. The isolates were from 9 locations: 8 districts in Uganda and a ninth from patients who acquired their illness in Juba, South Sudan, and were treated in Uganda. All 63 isolates tested positive for *ompW*, *toxR* and *ctxA* indicating the presence of *V*. *cholerae* virulence genes. The isolates included both *V*. *cholerae* Inaba (63%) and Ogawa (34%) serotypes as shown in Table 1.

**Table 1:** District of origin, number of isolates by year of isolate identification and serotype of *V*. *cholerae* isolates tested using PCR, MLVA and WGS.

Location	Number of isolates by year of isolation			Serotype	Total
**District**	**2014**	**2015**	**2016**		**2014–2016**
**Moyo**	12	0	0	Inaba	12
**Arua**	3	2	0	Inaba	5
**Hoima**	0	5	0	Inaba	5
**Kasese**	0	15	0	Ogawa & Inaba	15
**Kampala**	0	5^a^	1	Ogawa	6
**Mbale**	0	0	15	Ogawa	15
**Moroto**	0	1	0	Ogawa	1
**Mityana**	0	0	2	Ogawa	2
**Juba, South Sudan**	0	2	0	Inaba	2
**Total**	**15**	**30**	**18**		**63**

^a^—includes Wakiso district.

**Fig 1:**
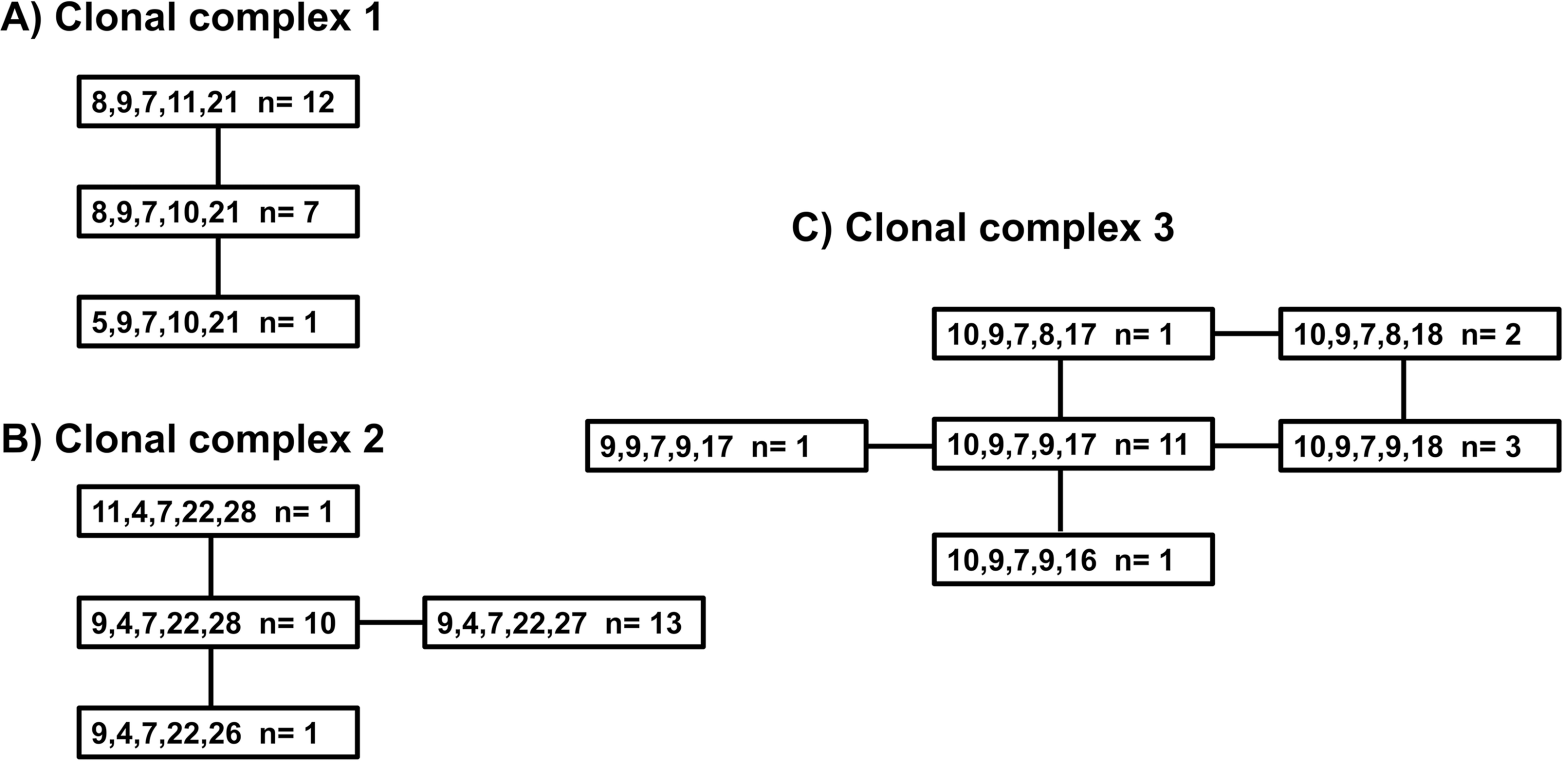
MLVA CC for *V*. *cholerae* associated with outbreaks in Uganda. Each genotype is represented by five numbers indicating the number of repeats at the five loci, VC0147, VC0436-7 (intergenic), VC1650, VCA0171 and VCA0283. ‘N = ‘ reports the number of isolates with that genotype. The lines connecting the boxes indicate variation at a single locus. Part A is Clonal Complex 1, Part B is Clonal Complex 2, and Part C is Clonal Complex 3.

All 63 *V*. *cholerae* isolates were genotyped using MLVA. Three clonal complexes (CC) were identified circulating in Uganda. MLVA CC1 contained 32% (20/63); MLVA CC2, 40% (25/63); and MLVA CC3, 28% (18/63) of the isolates. The three MLVA CCs are shown in Fig 1.

**Fig 2:**
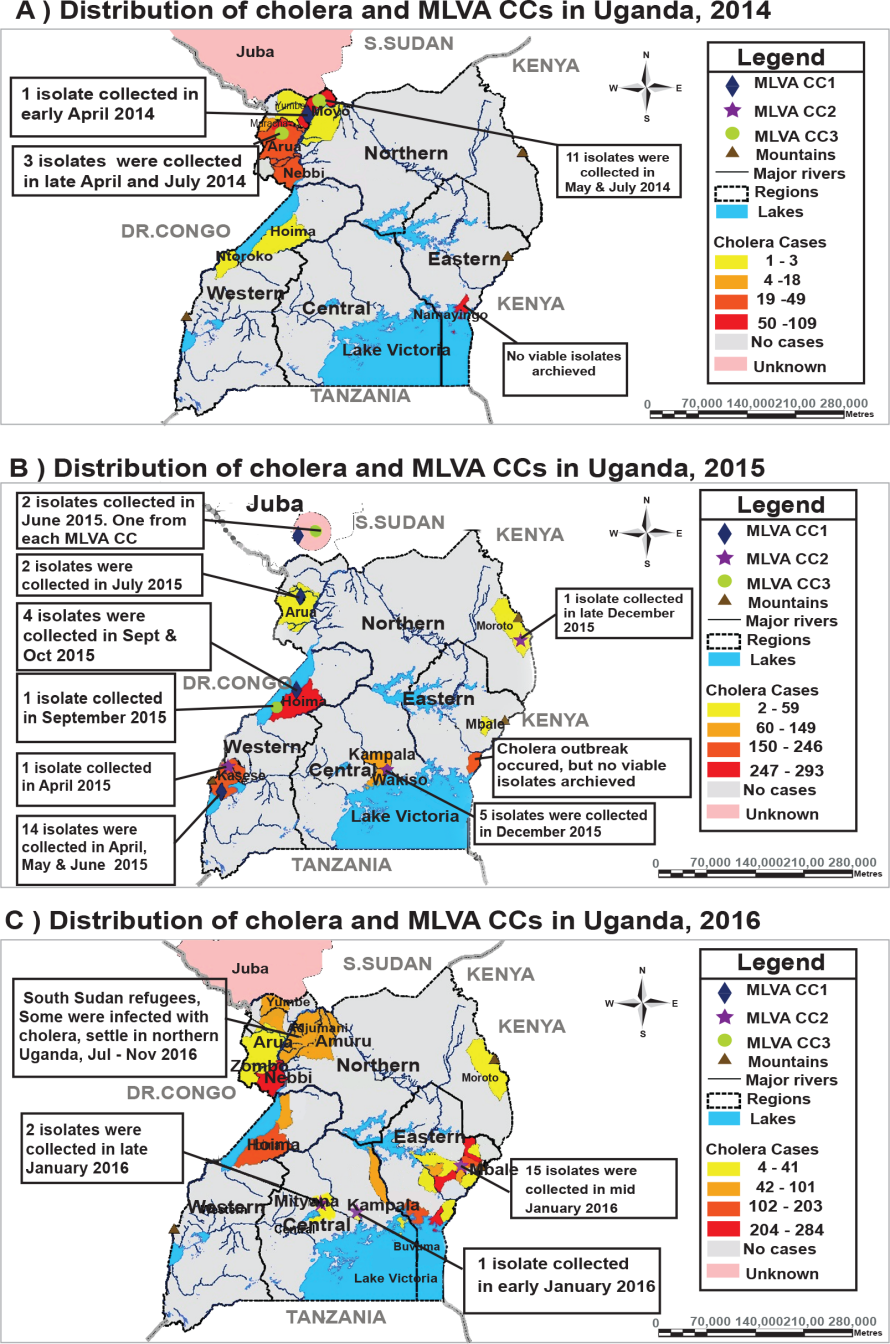
Shows the spatial distribution of MLVA clonal complexes and location of cholera outbreaks in Uganda: Part A during 2014, Part B during 2015, and Part C during 2016. Clonal Complex 1 are green circles, CC2 are purple stars, and CC3 are dark blue diamonds. The number of cases reported from each area varies by the year, yellow is the fewest number of cases, orange, then red-orange and red is the largest number of reported cases. Grey color indicates that there were no reported cases and blue indicates the Great Lakes of Africa.

The spatial distribution of MLVA CCs in Uganda reveals the presence of multiple genetic lineages within outbreaks and genetically defined connections between outbreaks (Fig 2). Two lineages were observed in 2014, when CCs 1 & 3 were isolated in Arua and Moyo districts in northwest Uganda. In 2015, CCs 1 & 3 were observed in Hoima and CCs 1 & 2 were isolated in Kasese district in southwest Uganda.

Each separate CC identified one of three genetically related series of outbreaks. First, isolates from CC3 were observed in June 2015 in individuals from Juba, South Sudan, and later in July 2015 in nearby Arua district, Uganda. Additional isolates were seen further south in September 2015 in Hoima on Lake Albert in Uganda. A second outbreak, defined by CC2, was initially identified in April 2015 in Kasese district in western Uganda, and subsequently in November 2015 in Wakiso district in central Uganda, in December 2015 in Kampala district in central Uganda and in December 2015 in Moroto district in northeastern Uganda. This outbreak persisted into January 2016 when it was found in Kampala and Mityana in central Uganda and in Mbale district in eastern Uganda. A third outbreak, defined by CC1, contained isolates collected in May and July 2015 in Kasese district, Uganda, and in June 2015 in individuals from Juba, South Sudan.

**Fig 3:**
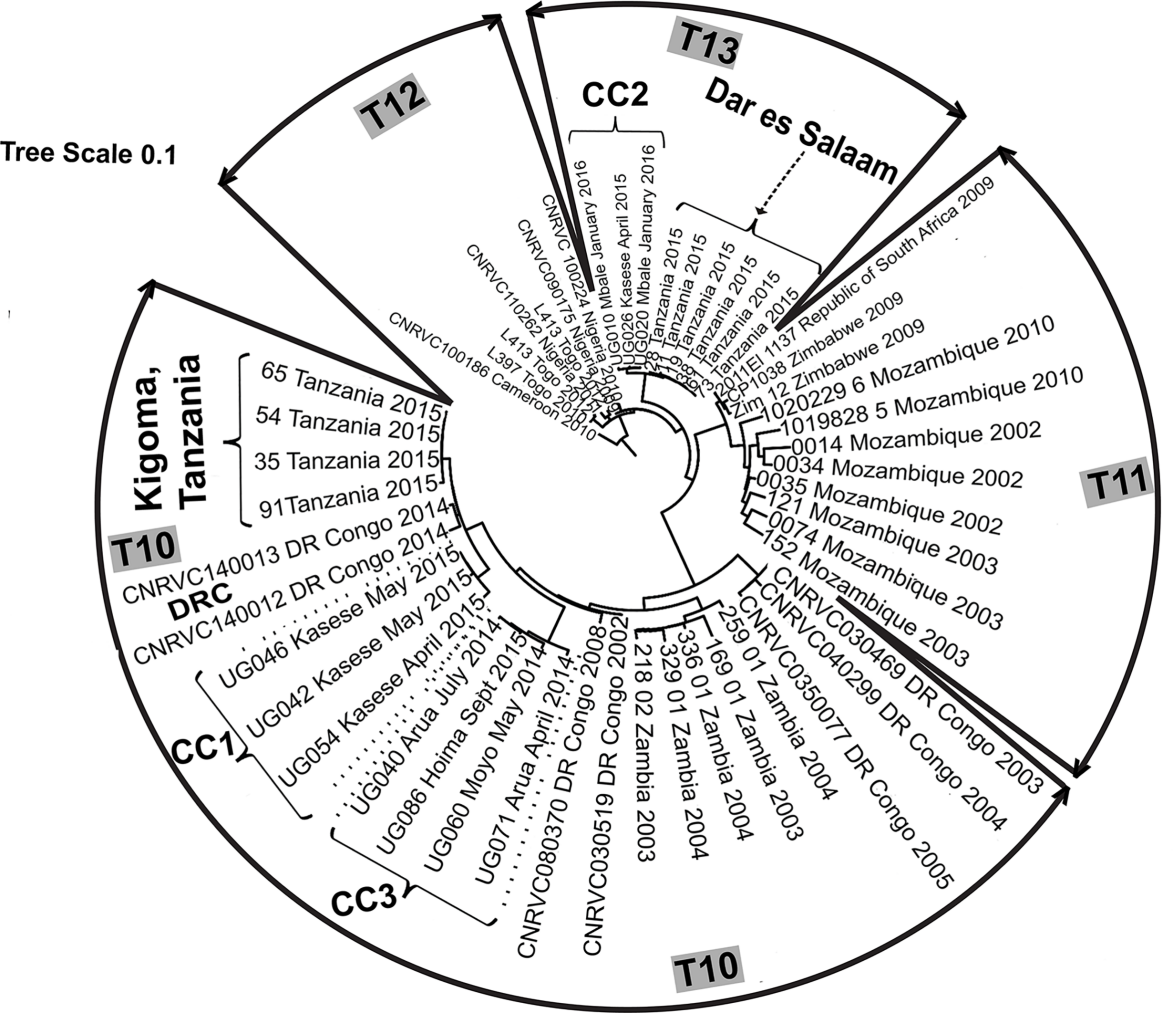
Phylogram of *V*. *cholerae* WGS data. Forty-one sequences from African isolates representing T10, T11 and T12 were included. Solid lines and black arrows demarcate the boundaries of the transmission events (T). Dotted lines and outlined arrows demarcate the boundaries of the clonal complexes (CC) in Uganda. Dashed arrows identify specific isolates from locations outside Uganda inferred to be examples of cross-border spread. The sequences within the Ugandan clades were less than five nucleotides apart. Those sequences in the Tanzanian clades were less than nine nucleotides from the Ugandan sequences of the closest clade. The radial lines are proportional to the number of nucleotide differences.

WGS genotyping of ten isolates indicated that the DNA was typical of the third wave of the seventh pandemic containing the classical allele of *ctxA* (S2 Table). The Ugandan DNA sequences belonged to three distinct clades. Within these distinct clades, the Ugandan sequences differed by five or fewer nucleotides (Fig 3). Two clades were contained in the transmission event (T)10 lineage and the other was contained in T13; no Ugandan isolate sequences were contained in a third African lineage T12.

The Ugandan clades were closely related to each other and to sequences from Democratic Republic of Congo and Tanzania (Fig 3). Clade 2 sequences from Kasese district in April 2015 were related most closely to sequences from Mbale district in January 2016 and secondarily to sequences from i) the Democratic Republic of Congo and ii) epidemic isolates from Dar es Salaam, Tanzania in August 2015 which spread across Tanzania during 2015. Clade 3 sequences from Arua and Moyo districts, Uganda in April and May 2014 and Clade 1 sequences from Kasese district, Uganda in April and May 2015 were related closely to sequences from an outbreak in January 2015 in Kigoma, Tanzania. The distance between the Ugandan and Tanzanian clades was nine or fewer nucleotides.

## Discussion

Our data are consistent with the spread of multiple genetic lineages of *V*. *cholerae* within Uganda and across its borders during 2014, 2015 and 2016. We found three CCs identified by MLVA that corresponded to the three clades of sequences by WGS. Each of these three genetic lineages displayed cross-border spread and spread within Uganda. The cross-border spread was both into and out of Uganda. These three clades circulating in East Africa belong to wave 3 of the seventh cholera pandemic, *ctx* carrying *V*. *cholerae* El Tor strain and belong to the T10 and T13 introductions of *V*. *cholerae* into East Africa [29].

Our data do not change the fundamental topology of the phylogenetic tree for *V*. *cholerae*. However, our WGS data revealed incidences of cross-border spread and of spread within Uganda. One example of cross-border spread was demonstrated by the close relationship between isolates (CC1, Clade 1, T10) from i) the Democratic Republic of Congo in 2014, ii) an outbreak in January 2015 in Kigoma, Tanzania, on the shores of Lake Tanganyika, iii) isolates from an outbreak in April and May 2015 in Kasese district on the western border of Uganda about 600 kilometers north of Kigoma, and iv) extended based on MLVA data to include the travelers seeking medical care in Uganda from, Juba, South Sudan. Cross-border spread between the Democratic Republic of Congo, South Sudan and Uganda was previously inferred from epidemiological evidence alone [30,31]. A second cross-border spread was revealed by the close relationship between isolates from an outbreak (CC2, Clade 2, T13) in April 2015 in Kasese district and those from Dar es Salaam, Tanzania, in August 2015 [32]. This lineage also spread from Kasese district to Mbale district in January 2016 or perhaps the seeding of these early 2016 cases came from Tanzania. The genetic distances between the various isolates was too small for the origin to be determined with certainty. Although these two incidences of cross-border spread included isolates from Kasese district in April 2015, the isolates that spread were from two distinct genetic lineages. This finding implies that the two distinct genetic lineages were present at the same time in the cholera outbreak in Kasese district similar to the cholera outbreak in Kenya in January 2009 –May 2010 in which two distinct lineages were also found [33]. A third example of cross border spread comes from MLVA CC3 (Clade 3, T10), the genetically related isolates included isolates from Kigoma, Tanzania and Kasese district, Uganda in January and April 2015. Additional isolates were collected in June 2015 among the fishing community in Hoima district on Lake Albert, Uganda indicating spread within Uganda. A fourth example of cross-border spread comes from the presence of South Sudanese refugees in Uganda in the last half of 2016 seeking health care for cholera, although no isolates were available for testing.

Examples of spread within Uganda included CC3 that was found in April and May 2014 in Arua and Moyo districts respectively, 125 kilometers apart, in northwest Uganda; and was found in July 2015 again in Arua district and in September 2015 in Hoima district, 250 kilometers to the southwest. A second example of spread within Uganda is CC2, initially identified in Kasese district in April 2015 and identified subsequently in December 2015 in Kampala and Moroto districts, in central and eastern Uganda respectively, although the latter could have come from Tanzania, as the genetic data are insufficient to distinguish between the two alternatives.

Tracking the spread of *V*. *cholerae* requires genetic identification as demonstrated by the presence of multiple genetic lineages occurring simultaneously in the same region. Multiple lineages were collected in Moyo, Kasese and Hoima districts in Uganda. Multiple lineages were found despite our analyses being limited to a small number of isolates.

Analyses of additional isolates may identify even more cases of multiple lineages in a single location. Each genetic lineage in a given location probably represents an independent introduction event to that location. The caveat to that hypothesis are the reports of multiple lineages within a single person [18], a phenomenon that has not been explored in Africa.

The spread of cholera inferred by this study is consistent with the documented movement of populations including refugees and traders affecting communities located along the great lakes, rivers, fishing villages, and trade and communication routes [30,34]. This is supported by evidence from the 2016 cholera outbreak in northern Uganda that was confined to districts hosting refugees from or bordering South Sudan.

These findings have several implications for cholera control in the region. Apart from providing a baseline for future molecular studies in Uganda, they demonstrate the need for approaches to disease prevention and control that cross national boundaries. In addition to strengthening interventions within countries, an approach similar to that taken to contain Ebola in West Africa [35,36] should be adopted. An outbreak in one country should elicit support from neighbors to ensure timely control [37]. Cross-border collaboration and joint interventions between neighboring countries should be implemented and sustained over an extended period to promote cholera elimination.

### Study limitation

No *V*. *cholerae* isolates were collected and tested from a cholera outbreak in 2016 in northwestern Uganda that started with the influx of South Sudan refugees and was restricted to districts where the refugees settled and their immediate neighborhoods. However, since this outbreak was restricted to a few districts in northwestern Uganda with refugees, it is unlikely that this had an effect on the findings of this study.

### Conclusion

The cholera outbreaks in Uganda were due to genetically diverse *V*. *cholerae* O1 isolates from two introductions from wave 3 of the seventh pandemic carrying the classical El Tor toxin gene. The *V*. *cholerae* strains showed local and regional transmission within Uganda and East Africa. Interventions to prevent, control, and eliminate cholera in Uganda and throughout East Africa should be strengthened with a focus on regional collaboration.

## Supporting information

S1 Checklist STROBE checklist for cross-sectional studies. (PDF)

S1 Table Genotyping results for Uganda *V*. *cholerae* isolates and location of genetic data. "The genomes were submitted to NCBI and accessible under the BioProject ID PRJNA439310." Annotated genomes are available at Genbank accession numbers PYRD00000000-PYRM00000000.
(XLSX)

S2 Table DNA sequences used in analysis of the African *V*. *cholerae* strains. (XLSX)

S1 Dataset Epidemiological data on *V*. *cholerae* outbreaks in Uganda that was used in production of the maps. (XLS)
